# Does Polycystic Ovary Syndrome Itself Have Additional Effect on Apelin Levels?

**DOI:** 10.1155/2014/536896

**Published:** 2014-10-07

**Authors:** Dilek Benk Silfeler, Cumali Gokce, Raziye Keskin Kurt, Nigar Yilmaz Atilgan, Oktay Hasan Ozturk, Ebru Turhan, Ali Baloglu

**Affiliations:** ^1^Department of Obstetrics and Gynecology, Faculty of Medicine, Mustafa Kemal University, Antakya, Hatay, Turkey; ^2^Department of Internal Medicine, Faculty of Medicine, Mustafa Kemal University, Antakya, Hatay, Turkey; ^3^Department of Family Medicine, Faculty of Medicine, Mustafa Kemal University, Antakya, Hatay, Turkey; ^4^Department of Public Health, Faculty of Medicine, Mustafa Kemal University, Antakya, Hatay, Turkey

## Abstract

*Objective*. The present study was designed to compare serum levels of apelin between lean PCOS women and healthy women with regular menses. *Study Design*. A total of 30 lean patients with PCOS and 30 healthy subjects were included in this study. Serum apelin levels were compared between groups. *Results*. Serum apelin levels in lean PCOS patients were not significantly different from the control subjects. *Conclusion*. Our findings indicate that PCOS itself does not seem to change apelin levels. Further investigation on a large number of subjects will need to be conducted to prove the consistent or variable association in PCOS.

## 1. Introduction

Polycystic ovary syndrome (PCOS) is a common endocrinologic disorder seen in 4–8% of women of reproductive age [1]. PCOS is characterized by hormonal and metabolic abnormalities. This syndrome is associated with increased risk of type 2 diabetes, dyslipidemia, and other cardiovascular diseases. The main characteristic features of the syndrome include chronic anovulation, hyperandrogenism, and insulin resistance [1–4]. The underlying mechanism of metabolic disorders in PCOS has not been completely understood yet. Even then, central obesity appears as an important factor to increase the risk of metabolic abnormalities in PCOS [4, 5]. However, it has been shown that the insulin resistance and metabolic disorders are also seen in lean PCOS women [4]. Insulin resistance and inflammation are associated with dysfunctional secretion of adipokinesin both of the obese and nonobese PCOS patients [6].

Apelin, which is one of the new described adipokines, is secreted by mature adipocytes in human [7, 8]. Apelin plays a key role in the regulation of normal glucose and lipid metabolism and it is associated with insulin resistance [7]. It has been reported that a positive correlation exists between plasma apelin levels and body mass index (BMI) [9].

In animal experiments, it is shown that apelin secretion by adipocytes and plasma apelin levels increase in hyperinsulinemia related obesity [9]. The activated apelinergic system was also found in patients with hypertension, heart diseases, obesity, glucose intolerance, and type 2 diabetes mellitus [7, 10–13]. In most of researches, plasma apelin levels were found lower in PCOS patients [7].

The aim of the study was to compare serum levels of apelin between 30 women with lean PCOS and controls.

## 2. Material and Methods

The study population consisted of 30 PCOS patients and 30 body mass index (BMI) and age matched healthy subjects as a control group from the Outpatient Clinic of Obstetrics and Gynaecology Department. PCOS was diagnosed according to the Rotterdam group criteria in which patients should have at least two of three features (oligo- or amenorrhea, clinical and/or biochemical signs of hyperandrogenism, and ultrasonographic appearance of the polycystic ovary) after excluding other etiologies including hyperprolactinemia and nonclassical adrenal hyperplasia [14]. BMI was calculated as weight divided by the square of the height (kg/m^2^). Lean patients were defined as BMI from 18.5 to 24.9 kg/m^2^. Patients with hypertension, hyperprolactinemia, diabetes mellitus (DM), thyroid disease, BMI of ≥25 kg/m^2^, and other systemic diseases were excluded. The study is approved by the local ethical committee of Mustafa Kemal University.

All the participants underwent a detailed physical examination. Hirsutism scores were determined according to modified Ferriman-Gallwey scoring system (>8 points mean clinical hyperandrogenism) [15]. BMI, systolic and diastolic arterial blood pressure, waist circumference (cm), antral follicle count, and ovarian volume with transvaginal ultrasonography were evaluated. We measured the longitudinal size and transverse diameter of ovaries. After turning transducer 90 degrees, anteroposterior diameter was measured where it is the thickest diameter. We calculated the volume of uterus and ovary by this formula (*V* = *D*1 × *D*2 × *D*3× 0,523) whether to accept these organs ellipsoid (*D*1 is transvers, *D*2 is anteroposterior, and *D*3 is longitudinal diameter) [16].

In PCOS group, after 12 hours of fasting on the third day of menstrual cycle, standard oral glucose tolerance test, the early follicular phase hormone profile, triglyceride, high density lipoprotein (HDL), low density lipoprotein (LDL), very low density lipoprotein (VLDL), blood glucose, insulin, hemoglobin a1c (HbA1c) levels, and serum levels of apelin were measured. In control group, we performed the same tests except oral glucose tolerance test. Hormone profile included thyroid stimulating hormone (TSH), follicle stimulating hormone (FSH), luteinizing hormone (LH), prolactin (PRL), estradiol (E2), free testosterone, total testosterone, androstenedione (AS), 17-hydroxyprogesterone (17-OHP), dehydroepiandrosterone sulphate (DHEAS), and sex hormone binding-globulin (SHBG). They were measured using standard enzymatic methods with a fully automated random access chemiluminescence enhanced enzyme immunoassay system (Roche Laboratory Systems, Mannheim, Germany). Plasma glucose level was determined by the glucose oxidase method. Plasma insulin was measured by electrochemiluminescence immunoassay (Roche Diagnostic GmBH, Mannheim). Serum glucose level was measured after a 75-gram oral glucose load in oral glucose tolerance test. Insulin resistance was calculated according to homeostatic model assessment of insulin resistance (HOMA-IR) formula [HOMA-IR = fasting insulin (mIU/mL) × fasting glucose (mg/dL)/22.5]. Plasma lipid profiles were analyzed by LX-20 Pro chemistry analyzers (Beckman Coulter).

The blood samples were centrifuged to separate the serum and stored at −80°C until used for the apelin assays. Serum apelin levels were analyzed by a human/mouse/rat apelin C-terminus enzyme immunoassay kit. The intra- and interassay coefficients of variation were of 10% and 15% for apelin.

### 2.1. Statistical Analysis

SPSS v.15.0 program was used for statistical analysis of the study. Independent sample *t*-test and Levene's test were used for the comparison. *t*-test for independent samples and Pearson's correlation analyses were used in statistical evaluation of the data. Results were given as mean ± standard deviation using the standard deviation from average of central tendency measures and measures of change. All the results were considered statistically significant with a *P* value of <0.05.

## 3. Results

The characteristics of 30 lean patients with PCOS and 30 healthy controls are summarized in Table 1. There was no significant difference between the groups in terms of BMI, age, systolic and diastolic blood pressure (mmHg), fasting blood glucose, HbA1C, fasting insulin, HOMA-IR, HDL, LDL, and total cholesterol, TSH, FSH, LH, E2, PRL, total testosterone (TT), free testosterone (fT), AS, 17-OH progesterone (17-OHP), DHEAS, and SHBG levels (Table 2). However, the triglyceride level was significantly higher in patients with PCOS than control group (74.73 ± 31.03 versus 60.53 ± 16.31; *P*: 0.03). There was no statistical difference between PCOS patients and control subjects with regard to serum apelin levels (5.2 ± 12.1 ng/mL versus 6.7 ± 8.3 ng/mL; *P*: 0.595).

Each ovary volume and ovarian follicle counts were significantly higher in patients with PCOS than control subjects (*P* < 0.05) (Table 2). Oral glucose tolerance test (OGTT) was performed on all the individuals with PCOS and all the cases had normal results.

The correlation analysis between apelin levels and other parameters (HbA1c, fasting insulin, HOMA-IR, HDL, LDL, total cholesterol (T-Chol), triglycerides, TSH, FSH, LH, PRL, E2, TT, fT, AS, 17-OHP, DHEAS, and SHBG levels) is shown in Table 2. No correlation was found between the apelin levels and all the other parameters in the groups.

## 4. Discussion

In the present study, we compared the serum apelin levels between lean PCOS patients and the healthy individuals. Serum apelin levels in lean PCOS patients were not significantly different from the control subjects.

Chang et al. revealed that the level of apelin in PCOS patients is lower than the control subjects [7]. In that study, both obese and nonobese cases were evaluated. In contrast to this finding, in Cekmez et al.'s study, the mean level of apelin was found higher in adolescents with PCOS than control group and a positive correlation was reported between the apelin levels and BMI and HOMA-IR [17]. Obese adolescents with PCOS were evaluated in their study. However, in our study, patients with lean PCOS were enrolled, and the mean BMI of these patients was lower than the PCOS cases of other previous two studies. We think that our results were different from these two researches because of age and BMI differences.

In the current study, although the levels of apelin were lower in PCOS group than control group, this difference was not significant. It is proposed that our results were different from these two researches because of age and BMI differences. These data sets were divergent from the results presented in Olszanecka-Glinianowicz et al.'s study. Different ethnicity, age, and fat distribution in the studied groups were a weak explanation for the observed changes [18]. Olszanecka-Glinianowicz et al. suggested that the increased apelin levels observed in normal weighted PCOS women related to insulin resistance. That increase in plasma apelin levels in lean PCOS subjects was disturbed in obese PCOS women. The levels were decreased in obese but increased in lean PCOS when compared to non-PCOS subjects [18]. The implication of apelin in the pathogenesis of PCOS seems to be more complicated, involving disturbed synthesis of androgens. Plasma levels of both apelin isoforms in their study correlated positively with serum concentrations of androstenedione [18]. However, in this prospective study, although apelin level is increased, AS is decreased. Despite the correlation between apelin and AS, there is no statistical difference.

Choi et al. pointed that serum apelin levels were lower in nonobese women with PCOS compared with controls and levels of apelin were correlated only with androgen status. These findings may have indicated that women with lean PCOS were at high risk for CVD, which was further aggravated by hyperandrogenism [19]. The results of current study demonstrate that androgen levels in lean PCOS patients were not significantly different from the control group.

In conclusion, our findings suggest that apelin is involved in the pathogenesis of PCOS-associated insulin resistance, but there was no significant difference in apelin levels of lean PCOS patients and control subjects. Secretion of apelin is strongly inhibited by fasting whereas its secretion increases after feeding, similar to insulin. These findings suggest that the use of insulin provides direct control over apelin gene expression in adipocytes. It was shown that the levels of plasma apelin and insulin are very high in obese patients [20].

Apelin might play a role in the complicated metabolic abnormalities of the syndrome in obese women. Therefore, apelin levels may increase in obese PCOS women. If PCOS patients, without insulin resistance and BMI < 25, were evaluated, apelin levels would be similar in healthy women. PCOS itself does not seem to change apelin levels. Furthermore, the large series and new studies are needed to clarify this issue.

## Figures and Tables

**Table 1:** Demographic features of groups.

	PCOS (*n* = 30)	Control (*n* = 30)	*P*
BMI	20,76 ± 2,08	20,04 ± 2,22	0,199
Age	22,46 ± 4,11	23,66 ± 7,08	0,426
Age of menarche	13,43 ± 1,38	13,56 ± 1,38	0,710

**Table 2:** Biochemical and ultrasonographic comparison of groups.

	PCOS (*n* = 30)	Control (*n* = 30)	*P*
Apelin	5,23 ± 12,06	6,68 ± 8,34	0,595
Glucose	89,18 ± 6,82	92,26 ± 12,86	0,251
HbA1C	4,87 ± 0.50	5,08 ± 0,61	0,166
Insulin	9,87 ± 10,92	7,83 ± 4,08	0,340
HOMA-IR	2,28 ± 3,00	1,83 ± 1,21	0,442
TG	74,73 ± 31,03	60,53 ± 16,31	0,030∗
HDL	47,31 ± 12,91	48,79 ± 8,09	0,598
LDL	101,34 ± 29,90	94,68 ± 23,30	0,339
T.Col	163,63 ± 36,25	155,66 ± 25,23	0,327
TSH	1,68 ± 0,85	1,44 ± 0,71	0,241
FSH	5,58 ± 1,57	7,47 ± 5,13	0,059
LH	6,43 ± 4,02	5,64 ± 5,60	0,530
PRL	20,56 ± 10,20	17,74 ± 6,88	0,214
E2	37,73 ± 18,30	34,60 ± 18,62	0,514
*t*-test	3,28 ± 14,86	0,62 ± 0,22	0,333
*F* test	3,36 ± 1,49	4,87 ± 2,18	0,759
AS	4,87 ± 2,18	5,40 ± 1,75	0,312
17-OHP	1,33 ± 0,81	1,17 ± 0,67	0,429
DHEAS	259,80 ± 115,46	309,35 ± 129,23	0,123
SHBG	73,76 ± 47,33	82,95 ± 45,68	0,447
Vol. of right ovary	7747,19 ± 4006,46	5009,64 ± 3234,95	0,005∗
Vol. of left ovary	7632,11 ± 3932,68	4871,09 ± 2365,71	0,002∗

*Statistical significance (*P* < 0.05).
